# Repellent, oviposition-deterrent, and insecticidal activity of the fungal pathogen *Colletotrichum fioriniae* on *Drosophila suzukii* (Diptera: Drosophilidae) in highbush blueberries

**DOI:** 10.1038/s41598-020-71341-y

**Published:** 2020-09-02

**Authors:** Pablo Urbaneja-Bernat, Timothy Waller, Cesar Rodriguez-Saona

**Affiliations:** 1 Department of Entomology, Rutgers University, New Brunswick, NJ USA; 2 Rutgers University, Rutgers Cooperative Extension, Millville, NJ USA

**Keywords:** Agroecology, Entomology

## Abstract

Spotted-wing drosophila, *Drosophila suzukii*, and the anthracnose pathogen *Colletotrichum fioriniae* are an important insect pest and fungal disease of highbush blueberries, respectively, in the United States. However, whether *C. fioriniae* infection affects *D. suzukii* preference and performance remains unknown. Here, we conducted choice and no-choice studies to determine the repellent, oviposition-deterrent, and insecticidal effects of *C. fioriniae* on *D. suzukii*. In choice tests, blueberry fruit treated with anthracnose solutions containing spores from either field-collected infected fruit (‘fruit’) or a laboratory *C. fioriniae* culture (‘colony’) were less attractive to sexually mature *D. suzukii* females, but not males, than untreated fruit. The plant tissue (fruit or leaves) did not influence *C. fioriniae* repellency effects on *D. suzukii*. In no-choice tests, 55% fewer numbers of eggs were laid on, and 65% fewer adults emerged from, blueberry fruit treated with either the ‘fruit’ or ‘colony’ anthracnose solution than untreated fruit. Egg-to-adult *D. suzukii* survival was also 12% lower on *C. fioriniae*-infected fruit. No repellency or negative effects on survival were observed when *C. fioriniae* spores were filtered out of the solution. These findings will help efforts towards the discovery of microbial-derived repellent/oviposition-deterrent compounds that could be used in behavior-based management strategies for *D. suzukii*.

## Introduction

Spotted-wing drosophila, *Drosophila suzukii* (Matsumura) (Diptera: Drosophilidae), an insect native to Southeast Asia, has become a major pest of soft- and thin-skinned fruit crops, including raspberries, strawberries, blueberries, blackberries, and cherries^1^. This frugivorous pest has rapidly expanded its geographic range across multiple continents, which now includes many Asian, North and South American, and European countries^2–4^. In the United States, this insect was first detected in the Western states (California) in 2008 and quickly spread to other states; by 2011, it was found in most Northeastern states^2,3^. Several behavioral and morphological features in *D. suzukii*, such as its attraction to odors from ripening fruit^5,6^ and an enlarged, serrated ovipositor, enable gravid females to be attracted to, and oviposit on, fresh fruit in addition to overripe fruit^7^. These features, among others, have contributed to its worldwide pest status compared with most *Drosophila* species that prefer rotten fruits and are, thus, not considered pests.

Because plant–insect and plant-pathogen interactions share many features including adaptations and counter-adaptions and negative effects of invasive species on humans^8^, understanding complex plant-pathogen-insect interactions could help in the discovery of novel pest management strategies in agroecosystems. *Drosophila* spp., including *D. suzukii*, have associations with microbes (i.e., fungi and bacteria) that can be mutualistic (where both species benefit)^9^, commensal (where only one of the species benefits)^10^, or antagonistic (where one species has a negative effect on the other)^11^. For example, *D. suzukii* has a strong relationship with mutualistic yeasts that improve larval survival and development and increase oviposition on fruits^12^. One example is *Hanseniaspora uvarum*, the most abundant yeast present in the alimentary canal of wild *D. suzukii* populations^13^. This may partially explain the greater attraction of *D. suzukii* to *H. uvarum* odors than to odors from other yeasts^9^; this yeast when present on fruits also increases feeding by gravid females^14^. Additionally, *Drosophila suzukii* can facilitate infection by pathogens that often results in fruit rotting, reduction of yield, and loss of marketability^15^. Indeed, a variety of yeasts and bacteria that cause sour rot in grapes gain access to the fruit’s carbohydrate-rich mesocarp through the feeding and oviposition activities of *Drosophila* spp.^16^. However, fruit infection by fungal pathogens can also negatively affect *Drosophila* spp. For example, *D. suzukii* larval survival and adult size were significantly reduced when they were reared on raspberries infected by *Botrytis cinerea* Pers., and fruit infection by this fungus also reduced fly attraction and oviposition^11^. Therefore, odors from microbes associated with *D. suzukii* could serve as sources of attractive and repellent compounds to manipulate the fly’s behavior in agroecosystems^17,18^.

In the highbush blueberry (*Vaccinium corymbosum* L.) agroecosystem, anthracnose fruit rot is an important disease mainly caused by the fungal pathogen *Colletotrichum fioriniae* (Sordariomycetes: Glomerellaceae)^19,20^, which can result in pre- and postharvest fruit losses^21–23^. The primary anthracnose symptoms are rotting of ripe fruit, both in the field before harvest and during storage after harvest, and the appearance of distinctive salmon-colored (conidial masses) droplets on the surface of rotting fruit^24^. If left untreated during bloom and if favorable (fungal) conditions persist, *C. fioriniae* infestation can result in significant crop losses^23,25,26^. *Colletotrichum* spp. infection is thought to speed the conversion of acids to sugars as a defense response in blueberries^27^ because there is a positive correlation between sugar concentration in fruit and fruit rot resistance^28^. This “overripe” effect of the pathogen on blueberry fruit could stimulate avian and mammalian herbivory^29–31^ and also have a positive effect on *D. suzukii* by increasing adult survival, longevity, and oviposition^7,32–34^. In fact, *D. suzukii* adults have a strong bias towards carbohydrate‐rich foods^35^. However, the Chinese chestnut anthracnose pathogen can cause high mortality of the Asian chestnut gall wasp *Dryocosmus kuriphilus* Yasumatsu^36^, indicating that interactions with anthracnose might not be beneficial to insects.

In this study, we tested two opposing hypotheses. First, given that *C. fioriniae*-infected fruit are expected to have more sugars, we hypothesized that *D. suzukii* females are more attracted to, and oviposit more eggs on, infected fruits than uninfected fruits (hypothesis 1). Alternatively, if *C. fioriniae* competes with *D. suzukii* for resources, we hypothesized that females are repelled by pathogen-fruit-associated odors and avoid ovipositing on infected fruits (hypothesis 2). To address this, we conducted choice and no-choice experiments to investigate the effects of *C. fioriniae*-infected fruit on *D. suzukii* attraction, oviposition, and immature performance. Specifically, we asked the following: (i) Are *D. suzukii* female and male responses to an attractive odor (blueberry fruit) affected by odors emitted from anthracnose (*C. fioriniae*)? (ii) Do anthracnose solutions from either infected fruit or a laboratory-grown *C. fioriniae* culture differentially affect *D. suzukii* adult attraction, oviposition preference, and offspring performance? (iii) Is the effect of *C. fioriniae* odors on *D. suzukii* influenced by the plant tissue it infects (fruit or leaf)? In other words, is the effect tissue-specific? (iv) Is there a relationship between *D. suzukii* adult preference and offspring performance on *C. fioriniae*-infested fruit? An understanding of this blueberry-*C. fioriniae*-*D. suzukii* association constitutes the first step towards the development of behavioral control strategies for *D. suzukii* based on odors from plant pathogens.

## Results

No symptoms, such as the distinct salmon-colored droplets on the surface of fruits, were observed prior to experiments; thus, any behavioral effects of anthracnose on *D. suzukii* adults (i.e., attraction and oviposition) were likely driven mainly by chemical rather than visual cues. Symptoms of fruit infection were seen after 7.23 ± 0.28 and 7.05 ± 0.26 days in the 'fruit’ and ‘colony' treatments (*t* = 0.47; df = 34; *P* = 0.641); no symptoms were observed in the 'filtered' and 'control' treatments.

### Effects of *C. fioriniae*-infected fruit odors on *D. suzukii* behavior—choice tests

**Figure 1:**
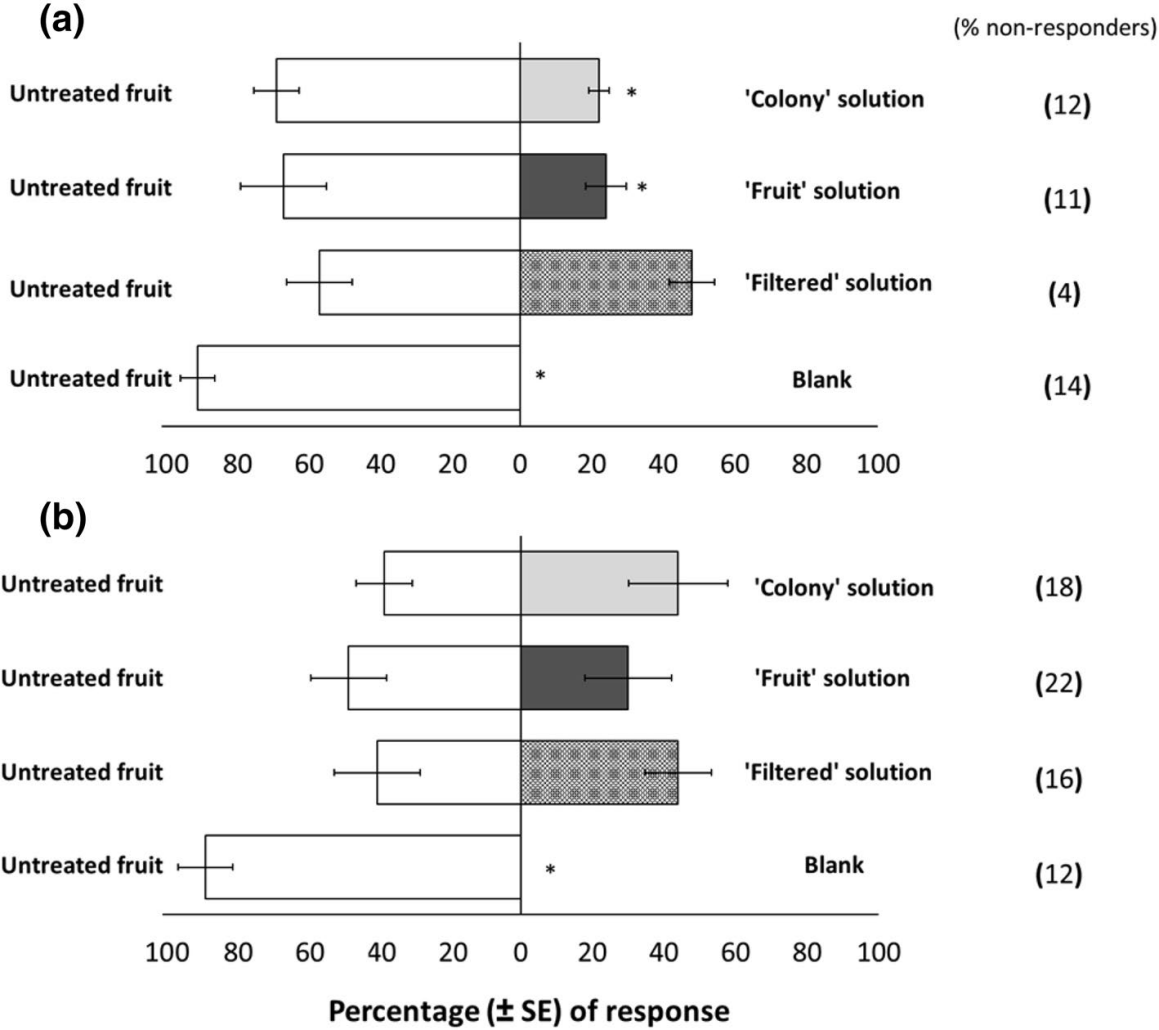
Sexually mature *Drosophila suzukii* female (**a**) and male (**b**) choice between highbush blueberry (*Vaccinium corymbosum*) fruit sprayed with four different treatments: (i) sterile ionized water (‘untreated fruit’) as a control, (ii) anthracnose solution from a laboratory *Colletotrichum fioriniae* culture (‘colony’), (iii) anthracnose solution from field-collected infected fruit (‘fruit’), and (iv) a solution prepared by filtering the ‘colony’ solution (‘filtered’) to remove the spores. In addition, flies were given a choice between highbush blueberry fruit versus no fruit (blank control). Percentages of nonresponders are shown in parenthesis. An asterisk (*) indicates significant differences between treatments, *P* ≤ 0.05.

Choice tests were conducted to determine the response of sexually mature *D. suzukii* females and males to anthracnose-infected blueberry fruit odors. When given a choice between blueberry fruit (without any fungal treatment) versus an empty vial (without fruit), 100% of the responding *D. suzukii* females and males were attracted to the fruit odors (females: *t* = 27.00, df = 9, *P* < 0.001; males: *t* = 16.5, df = 9, *P* < 0.001) (Fig. 1), showing that both sexes are attracted to odors from blueberry fruit. However, when given a choice between blueberry fruit treated with anthracnose solutions either from field-collected infected fruit (referred to as ‘fruit’) or from a laboratory *C. fioriniae* culture (‘colony’) versus untreated blueberry fruit, sexually mature *D. suzukii* females were 3 times more attracted to the untreated fruit than fruit treated with ‘fruit’ and ‘colony’ solutions (‘fruit’: *t* = 4.16, df = 9, *P* = 0.002; ‘colony’: *t* = 7.67, df = 9, *P* < 0.001). In contrast, no differences in *D. suzukii* female attraction were found when they were given a choice between fruits treated with a ‘filtered’ *C. fioriniae* solution (without conidia) and untreated fruit (*t* = 0.89, df = 9, *P* = 0.399) (Fig. 1a).

Compared to sexually mature females, *D. suzukii* males were not differentially attracted to blueberry fruit treated with the *C. fioriniae* ‘colony’ (*t* = 0.64, *df* = 9, *P* = 0.541), ‘fruit’ (*t* = 1.31, df = 9, *P* = 0.223), or ‘filtered’ (*t* = 0.38, df = 9, *P* = 0.716) solutions compared with the untreated fruit (Fig. 1b).

### Effects of plant tissue infected by C. fioriniae on *D. suzukii* behavior—choice tests

**Figure 2:**
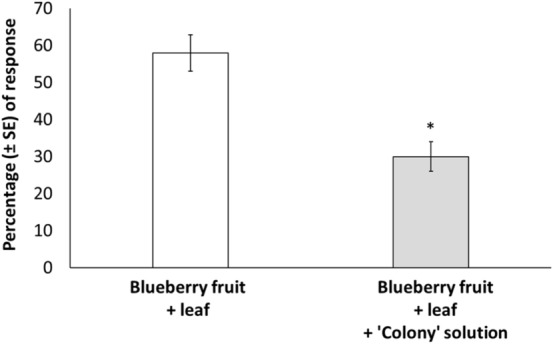
Sexually mature *Drosophila suzukii* female choice between highbush blueberry (*Vaccinium corymbosum*) fruit plus leaves sprayed with either sterile ionized water or an anthracnose (*Colletotrichum fioriniae*) solution (‘colony’). An asterisk (*) indicates significant differences between treatments, *P* ≤ 0.05.

To determine whether plant tissue influences the effects of *C. fioriniae* on *D. suzukii* behavior, we conducted additional tests in which sexually mature females were given a choice between blueberry fruit (food tissue) plus leaves (nonfood tissue) treated with *C. fioriniae* and fruit plus untreated leaves. *Drosophila suzukii* females were 2 times more attracted to the fruits with the untreated leaves than to fruits with the leaves treated with *C. fioriniae* (*t* = 3.28, df = 9, *P* = 0.010) (Fig. 2), indicating that the repellency effect of *C. fioriniae* on *D. suzukii* females was independent of the type of plant tissue.

### Effects of *C. fioriniae* infection on *D. suzukii* oviposition and adult emergence—no-choice tests

**Figure 3:**
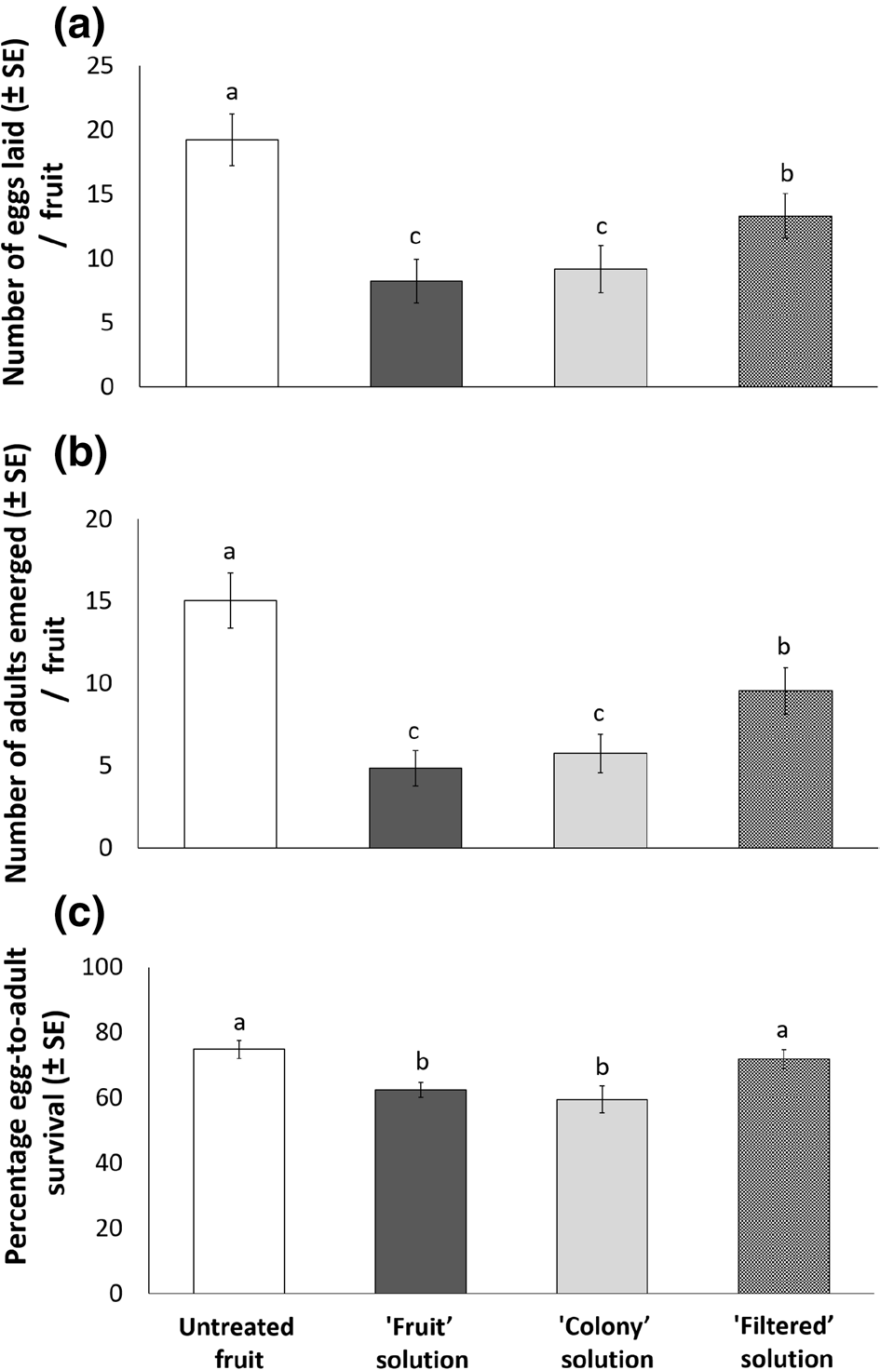
Effects on *Drosophila suzukii* oviposition (**a**), adult emergence (**b**), and percent egg-to-adult survival (**c**) of highbush blueberry (*Vaccinium corymbosum*) fruit sprayed with four different treatments: (i) sterile ionized water (‘untreated fruit’), (ii) anthracnose solution from a laboratory *Colletotrichum fioriniae* culture (‘colony’), (iii) anthracnose solution from field-collected infected fruit (‘fruit’), and (iv) a solution prepared by filtering the ‘colony’ solution (‘filtered’) to remove the spores. Different letters indicate significant differences among treatments according to Bonferroni pairwise tests.

A no-choice study was conducted to investigate the effects of *C. fioriniae* infection on *D. suzukii* oviposition, adult emergence, and percent egg-to-adult survival. Fifty-five percent fewer numbers of eggs were laid by *D. suzukii* on blueberry fruit treated with the *C. fioriniae* ‘colony’ and ‘fruit’ solutions than on the untreated fruit (*F*_3,80_ = 113.44; *P* < 0.001) (Fig. 3a). Thirty-one percent fewer eggs were also laid on fruit treated with the ‘filtered’ solution than on the untreated fruit (Fig. 3a). Sixty-five percent fewer *D. suzukii* adults emerged from blueberry fruit infected by the *C. fioriniae* ‘colony and ‘fruit’ solutions than from the untreated fruit (*F*_3,80_ = 120.718, *P* < 0.001) (Fig. 3b). Also, thirty-seven percent fewer adults emerged from the ‘filtered’ solution than from the untreated fruit (Fig. 3b). The percentage egg-to-adult survival was 12% lower in blueberry fruit treated with the *C. fioriniae* ‘colony’ and ‘fruit’ solutions than in the ‘filtered’ solution and the untreated fruit (*F*_3,80_ = 26.22; *P* < 0.001) (Fig. 3c), indicating a negative effect of *C. fioriniae* conidia on *D. suzukii* immature performance. No difference in percent egg-to-adult survival was observed between the ‘filtered’ solution and the untreated fruit (Fig. 3c).

## Discussion

The current study documents four important features of the interactions between the invasive insect pest *D. suzukii* and the fungal pathogen *C. fioriniae* in highbush blueberries: (i) *C. fioriniae* odors reduce the attraction of sexually mature *D. suzukii* females (but not males) to attractive blueberry fruit odors, showing that these females are repelled by odors from anthracnose-infected fruit; (ii) the ‘fruit’ and ‘colony’ anthracnose solutions had similar repellent, oviposition-deterrent, and insecticidal activity on *D. suzukii* females, indicating that the fungal pathogen *C. fioriniae* is mainly responsible for these effects; (iii) removing the fungal spores (conidia) from the anthracnose solution (‘filtered’ solution) eliminated the repellent effects of *C. fioriniae* on female *D. suzukii*; and (iv) the effect of *C. fioriniae* on *D. suzukii* was independent of the plant tissue (fruit or leaf).

Sexually mature *D. suzukii* females were less attracted to odors from blueberry fruits when treated with the anthracnose solutions (‘fruit’ and colony’). There are three possible, not mutually exclusive, mechanisms for these inhibitory effects. First, volatiles emitted from *C. fioriniae* could directly be responsible for the observed repellency effects. In fact, fungal odors such as geosmin and 1-octen-3-ol^37,38^ have been shown to negatively affect *D. suzukii* host-seeking and acceptance behaviors. Using video observations, Wallingford and collaborators^37,38^ found that 1-octen-3-ol prevents *D. suzukii* flies from coming into contact with the fruit (i.e., repellent [noncontact] effects), whereas geosmin inhibits egg-laying behaviors (i.e., oviposition-deterrent [contact] effects). Second, anthracnose infection could indirectly induce changes in volatile emissions from blueberry fruit, making them less attractive to *D. suzukii*. Many pathogens successfully exploit their host plants by inducing chemical changes to avoid, circumvent, or silence the hosts’ detection and/or defense systems^39–41^. These pathogen-induced chemical changes could affect volatile emissions from the host plant^42,43^. Although it needs verification, the fact that we observed repellent effects of *C. fioriniae* on *D. suzukii* females regardless of the plant tissue (fruit or leaf) suggests that these effects might not be due to changes in fruit odors but to odors emitted from *C. fioriniae* itself. Third, *C. fioriniae* odors could camouflage the blueberry odors, i.e., masking effect, thus preventing *D. suzukii* flies from detecting (i.e., reduce antennal sensitivity) and/or responding to these otherwise attractive odors^44^. Although testing these mechanisms was beyond the scope of this study, work is underway to isolate and identify the behaviorally active compounds from *C. fioriniae*-infected blueberry fruit on *D. suzukii*. Despite that both *D. suzukii* males and sexually mature females were attracted to noninfected fruit odors, only females were repelled by *C. fioriniae* odors, indicating that these odors provide information to host-seeking females on the suitability of oviposition sites. Although not tested in this study, we expect these fungal odors to affect (repel) mated females more strongly than virgin females, since the former likely utilize fruit odors for oviposition^17^. According to Revadi et al.^45^, *D. suzukii* females start producing offspring 2.5 days after emergence when kept together with males of the same age. We kept female and male flies together for at least 3 days prior to bioassays, which means that most females were likely ready to start egg laying. Future studies are needed to investigate whether physiological status (e.g., mating status and degree of starvation) of *D. suzukii* females influences their response to anthracnose odors.

Despite our choice assays showing clear repellent effects of *C. fioriniae* on *D. suzukii* females, about 20% of the tested flies were not affected by the fungal odors. In our studies, two variables that were constant included the time of anthracnose incubation and its concentration. We used a *C. fioriniae* incubation period of 48 h because this time of exposure was previously used to test the susceptibility of blueberry species (*Vaccinium* spp.) to anthracnose^46^. Previous studies have also shown that fruit rot pathogens, *Colletotrichum* spp., can successfully infect blueberry fruit after only 10 to 12 h postinoculation^26,47^ and induce chemical changes in fruit as early as 24 h after incubation^27^. Moreover, in preliminary studies, we showed that field-collected anthracnose-infected fruit had levels of spore concentrations similar to those used in our experiment (1.0 × 10^7^ per mL). Although further studies are needed to determine if increasing the time of anthracnose incubation or reducing the spore concentration affects *D. suzukii* behavior, our study documents for the first time the repellent effects of *C. fioriniae* under realistic conditions.

Under a no-choice scenario, *D. suzukii* oviposition was reduced in fruits infected by *C. fioriniae*. Anthracnose infection is known to increase sugar concentrations in fruits, possibly as a defense mechanism^27^ because there is a positive correlation between fruit sugar concentration and resistance to *Colletotrichum* spp.^28^. Given that *D. suzukii* females prefer to oviposit on fruit with high sugar concentrations due to their nutritional benefit^7^, we expected female oviposition preference for anthracnose-infected fruits (hypothesis 1). However, *D. suzukii* also prefers ovipositing on ripe fruit in which sugar concentrations are typically lower than in rotten fruit^48^. It is, thus, likely that *D. suzukii* oviposition preference is not determined solely by the sugar content in fruits^49^. Infection by many fungal pathogens, including *Colletotrichum* spp., typically leads to significant changes in the phytochemical composition of not just sugars but also alcohols, pH, and antimicrobial compounds^31,50^. These changes may be collectively perceived by *D. suzukii* females when assessing the quality of fruits during oviposition. It is also possible that females used visual cues to avoid oviposition on anthracnose-infected fruits; however, fruits did not show any signs of spoilage 48 h after infection.

There was a positive correlation between adult preference and immature performance: females avoided fruit infected by *C. fioriniae* that were of lower quality for offspring survival. Certain fungi, such as the yeast *H. uvarum*, have a mutualistic association with *D. suzukii*^13^ and have positive effects on *D. suzukii* larval development and survival^12^. In contrast, our study showed an antagonistic (insecticidal) effect of the fungal pathogen *C. fioriniae* on *D. suzukii*. Previously, Graziosi and Rieske^36^ had also shown that infection by *Colletotrichum acutatum* increases the mortality of the Asian chestnut gall wasp *D. kuriphilus*. Raspberries infected by the fungus *B. cinerea* also repelled *D. suzukii* and reduced larval survival and adult oviposition and size^11^. Interestingly, our results showed that both anthracnose solutions (‘field’ and ‘colony’), but not the ‘filtered’ solution, have repellent and insecticidal effects on *D. suzukii*, indicating that removing *C. fioriniae* conidia eliminates these negative effects. However, some yet unknown components of the ‘filtered’ solution also had some negative effects on *D. suzukii* oviposition. Compared to the ‘colony’ solution that was simply *C. fioriniae*, the ‘fruit’ solution was composed of more than one fungal species, although *C. fioriniae* was by far the most abundant of all species (T.W., pers. observation). In fact, *C. fioriniae* is highly competitive and typically reduces the abundance of other fungal species^51^. Therefore, it is likely that *C. fioriniae* competes with *D. suzukii* larvae for resource when co-exiting in blueberry fruits, which supports our hypothesis 2. The fact that disease symptoms were visible in fruit treated with the ‘fruit’ and ‘colony’ solutions, but not with the ‘filtered’ solution, about a week after infection suggests that fruit became a less suitable host for *D. suzukii*, which takes ~ 10 days to develop from egg-to-adult at 25 °C^52^, as the infection progressed.

In conclusion, this study is the first to demonstrate the antagonistic association between the fungal pathogen *C. fioriniae* and the insect pest *D. suzukii*. We showed that *C. fioriniae* odors, likely emitted from the spores (conidia), elicit a repellent response in sexually mature *D. suzukii* females and that *C. fioriniae* infection of blueberry fruits reduces both oviposition and immature survival in this fly. Altogether, these findings open new opportunities for the potential use of odors from the fungus *C. fioriniae* to manage *D. suzukii* in fruit crops once the behaviorally active compounds are identified.

## Methods

### Plant material

Highbush blueberry fruit (*V. corymbosum*; cv. ‘Bluecrop’) from a field located at the Rutgers P.E. Marucci Center, Chatsworth, New Jersey (USA), were used for laboratory experiments. Three fruit clusters per bush were bagged at fruit set (i.e., green-pink fruit, early June) to prevent pesticide exposure and natural insect infestation and considering that fruits are susceptible to *D. suzukii* once they start to color^7^. Fruit was harvested during maturation (i.e., blue fruit) from mid-June through mid-July, placed in clear polyethylene bags, transported to the laboratory, and used for the experiments. For experiments with leaves, green, healthy, mature highbush blueberry leaves (‘Bluecrop,’ unknown age) were collected from greenhouse-grown potted plants. Prior to experiments, the surface of fruits and leaves were thoroughly cleaned with sterile deionized water and then dried at room temperature (~ 25 °C).

### Insects

*Drosophila suzukii* flies were obtained from a laboratory colony initiated in 2013 from flies that emerged from blueberry-infested fruit collected in Atlantic County, New Jersey. The colony was maintained at the Rutgers P.E. Marucci Center. Flies were reared on a standard *Drosophila* artificial diet^52,53^ in 50-mL polystyrene tubes (Fisher Scientific, Nazareth, PA, USA) with ~ 15 mL of diet and plugged with BuzzPlugs (Fisher Scientific) and were kept in an incubator (Percival Scientific, Perry, IA, USA) set at 25 ± 1 °C, 65 ± 5% relative humidity (RH), and 16:8 light:dark cycle (L:D). Male and female flies used in the experiments were 3–7 days old and, thus, were sexually mature^45^. Flies were removed from the colony ~ 5 h before the start of each experiment.

### Fungal culture and treatment preparation

The laboratory *C. fioriniae* culture consisted of a single spore isolate (isolate BB#10)^19^ collected in 2005 from infected blueberry fruit at the Rutgers P.E. Marucci Center and was stored on corn meal agar (Becton, Dickinson and Company, Sparks, MD). High-density sporulation of the isolate was induced by streaking conidia onto clarified V8 juice agar^54^ with no calcium carbonate and a higher concentration of agar (28 g/L)^19^. Cultures were incubated at 25 °C in the dark for at least 7 days before spore suspension preparation.

Anthracnose spore suspension treatments were prepared using two sources: field-collected infected fruit (referred to as ‘fruit’) and a laboratory *C. fioriniae* culture (referred to as ‘colony’; described above). For the ‘fruit’ treatment, blueberry fruit showing symptoms of anthracnose, i.e., salmon-colored (conidial masses) droplets on the surface of rotting fruit, were collected at the Rutgers P. E. Marucci Center and incubated in plastic containers (15 × 15 × 5 cm) at room temperature (~ 25 °C) to promote sporulation. Once conidia were abundant (salmon-colored conidial masses), fruit were placed into a plastic screen mesh filter and repeatedly submerged into sterile deionized water. Once conidia were liberated, the spore concentration was estimated using a hemocytometer and adjusted to 1.0 × 10^7^ spores per mL of deionized water immediately before the beginning of experiments. This concentration was chosen because it was similar to the concentration of spores from the field-collected infected fruit samples. For the ‘colony’ treatment, *C. fioriniae* spore suspensions were prepared by washing conidia from the high-density cultures into deionized water and adjusting the spore concentration to 1.0 × 10^7^ spores per mL of deionized water, as described for the ‘fruit’ treatment, immediately before their use in experiments. In addition, to remove the conidia from any extracellular enzymatic compounds, a ‘filtered’ solution was prepared by sterile filtering (Cameo 25 GAS, 0.22 µm; Osmonics Penang, Malaysia) the ‘colony’ spore suspension described above immediately before use (following methods by Waller et al.^19^). Treated fruit were left to incubate at room temperature for 48 h before use in choice and no-choice assays (see below) to allow the solutions to settle on the fruit surface; this anthracnose incubation period is sufficient to activate defensive genes and induce secondary metabolite production in blueberries^27^. A group of 5 fruit from each treatment was set aside to confirm *C. fioriniae* infection by checking for symptoms (only fruit treated with the *C. fioriniae* ‘fruit’ and ‘colony’ solutions were symptomatic).

### Effects of *C. fioriniae*-infected fruit odors on *D. suzukii* behavior—choice tests

Choice assays were conducted to investigate the effects of odors from *C. fioriniae*-infected fruit on *D. suzukii* female and male behavior. Flies were given a choice between untreated fruit (control) and fruit treated either with the ‘fruit’ solution, ‘colony’ solution, or ‘filtered’ solution. In addition, flies were given a choice between untreated fruit and a blank control to determine their attraction to fruit in the absence of other odors. The experimental setup followed similar methods used by Feng and collaborators^55^. Choice arenas consisted of clear plastic cups (946 mL, 114-mm diameter, 127-mm height; Paper Mart, CA, USA). The lid of each cup had an 80-mm diameter circular hole cut in it, which was covered with a nylon mesh (anti-thrips insect screen, mesh size: 81 × 81; BioQuip, CA, USA) to provide ventilation while retaining flies. Polystyrene tubes (95 mm × 28.5 mm; same as those used for insect rearing) were labeled for the treated and untreated fruit. Equal quantities (~ 5 g) of blueberry fruit (*N* = 5) were used for all treatments, and fruits were placed inside of each tube. Fruits were sprayed with their corresponding treatment until run-off (approx. 1 mL of solution per fruit); untreated fruits were sprayed with deionized water only. Each pair of tubes in each choice test was sealed with Parafilm® (Pechiney Plastics Packaging Inc., Menasha, WI, USA), with a 4-mm-diameter hole in the center of the Parafilm to provide an entrance for the flies, and placed vertically on opposite sides of a cup. A cotton wick moistened with deionized water was placed on the bottom of each cup as a water source for the flies during the experiment. Ten *D. suzukii* (males or females) were released inside each cup (choice arena), and each choice test was replicated 10 times (*N* = 100 flies tested per choice test and sex). Testing flies in groups does not appear to affect their behavior in previous studies using similar choice tests^55^. The number of *D. suzukii* flies inside and outside the tubes in each choice arena was counted after 24 h. The experiment was carried out under a fume hood at 25 ± 1 °C, 60 ± 5% RH, and 16:8 h L:D.

### Effects of plant tissue infected by *C. fioriniae* on *D. suzukii* behavior—choice tests

We conducted choice tests to determine if the response of *D. suzukii* to *C. fioriniae*-infected fruit odors was mediated by changes in fruit odors. To address this, females were given a choice between blueberry fruit (food tissue) plus leaves (nonfood tissue) treated with the ‘colony’ solution versus fruit plus untreated leaves. Note that *C. fioriniae* primarily affects fruits but can also attack all other aboveground parts, including leaves. Only females were used in these experiments because males did not respond to *C. fioriniae* odors (see “Results” section). The same experimental setup as described before was used for these experiments. Each choice arena consisted of (i) a tube with blueberry fruit (~ 5 g; *N* = 5) plus leaves (~ 2 g; *N* = 10) treated with “colony” solution and (ii) a tube containing the same amount of fruit and leaves but the leaves were treated with deionized water only. Leaves were sprayed with their respective treatment until run-off. Ten *D. suzukii* females were placed inside each choice arena, and the experiment was replicated 10 times (*N* = 100 flies tested). The experiment was conducted under a fume hood under the conditions described above. The number of *D. suzukii* females inside and outside the tubes within the arenas was counted after 24 h.

### Effects of *C. fioriniae* infection on *D. suzukii* oviposition and adult emergence—no-choice tests

No-choice studies were conducted to determine the effects of *C. fioriniae* infection on *D. suzukii* oviposition and adult emergence. Blueberry fruit were placed individually in 118-mL (4 oz) translucent plastic cups (Solo Cup Co., Highland Park, IL, USA) and simultaneously exposed to one *D. suzukii* sexually mature female. Each fruit was treated with one of the four treatments described above: fruit’ solution, ‘colony’ solution, ‘filtered’ solution, or untreated control. A cotton wick moistened with deionized water was placed on the bottom of the plastic container as a water source. After 24 h of exposure, *D. suzukii* females were removed and all eggs laid in the fruit were counted under a stereomicroscope. Note that *D. suzukii* eggs are visible on the surface of fruit skins by observing the oviposition hole and two white breathing filaments protruding out of the egg^7^. All fruit containing eggs were subsequently stored in the cups with filter paper at room temperature (~ 25 °C). The number of emerged adults was recorded after14 days, which allowed enough time to complete egg-adult development^56^. As a measure of immature performance, for each fruit, we calculated the percent egg-to-adult survival by dividing the number of emerged adults by the number of eggs laid multiplied by 100. Each treatment was replicated 25 times.

### Data analyses

In choice experiments, for each cup (arena), data on the total number of *D. suzukii* flies inside each tube were recorded and the differences in *D. suzukii* response between treatments compared using paired *t*-tests. Flies that did not make a choice were considered as nonresponding and were not included in the statistical analysis. Choice data are shown as percentages in Figures.

In no-choice experiments, the effect of treatments on *D. suzukii* oviposition, adult emergence, and percent egg-to-adult survival (immature performance) was analyzed using generalized linear models (GLMs), followed by Bonferroni post-hoc tests. For oviposition and adult emergence, we used a Poisson distribution with a logit-link function. To analyze the percentage egg-to-adult survival, data were first arcsine transformed, and then analyzed using a normal distribution with identity link function. All statistical analyses were performed using SPSS Statistics 23.0 (IBM Corp, Armonk, New York, USA).
